# Determining Which Combinatorial Combat-Relevant Factors Contribute to Heterotopic Ossification Formation in an Ovine Model

**DOI:** 10.3390/bioengineering11040350

**Published:** 2024-04-03

**Authors:** Richard T. Epperson, Brad M. Isaacson, David L. Rothberg, Raymond E. Olsen, Brooke Kawaguchi, Ryan M. Rasmussen, Mary Dickerson, Paul F. Pasquina, John Shero, Dustin L. Williams

**Affiliations:** 1 Department of Orthopaedics, University of Utah, Salt Lake City, UT 84108, USA; brad.isaacson.ctr@usuhs.edu (B.M.I.); david.rothberg@hsc.utah.edu (D.L.R.); rayboss@gmail.com (R.E.O.); brooke.kawaguchi@utah.edu (B.K.); ras.ryan32@hotmail.com (R.M.R.); dustin.williams@utah.edu (D.L.W.); 2 Bone & Biofilm Research Laboratory, University of Utah, Salt Lake City, UT 84112, USA; 3 The Center for Rehabilitation Sciences Research, Uniformed Services University, Bethesda, MD 20814, USA; paul.f.pasquina.civ@health.mil (P.F.P.); john.c.shero.civ@health.mil (J.S.); 4 The Geneva Foundation, Tacoma, WA 98402, USA; 5 Office of Comparative Medicine, University of Utah, Salt Lake City, UT 84112, USA; mary.dickerson@gmail.com; 6 Department of Rehabilitation, Walter Reed National Military Medical Center, Bethesda, MD 20910, USA; 7 Extremity Trauma Center of Excellence, Joint Base San Antonio Fort Sam Houston, San Antonio, TX 78240, USA; 8 Department of Pathology, University of Utah, Salt Lake City, UT 84112, USA; 9 Department of Biomedical Engineering, University of Utah, Salt Lake City, UT 84112, USA

**Keywords:** heterotopic ossification, ectopic bone, trauma, large animal model, undecalcified histology, backscatter electron imaging

## Abstract

Traumatic heterotopic ossification (HO) is frequently observed in Service Members following combat-related trauma. Estimates suggest that ~65% of wounded warriors who suffer limb loss or major extremity trauma will experience some type of HO formation. The development of HO delays rehabilitation and can prevent the use of a prosthetic. To date there are limited data to suggest a standard mechanism for preventing HO. This may be due to inadequate animal models not producing a similar bone structure as human HO. We recently showed that traumatic HO growth is possible in an ovine model. Within that study, we demonstrated that 65% of sheep developed a human-relevant hybrid traumatic HO bone structure after being exposed to a combination of seven combat-relevant factors. Although HO formed, we did not determine which traumatic factor contributed most. Therefore, in this study, we performed individual and various combinations of surgical/traumatic factors to determine their individual contribution to HO growth. Outcomes showed that the presence of mature biofilm stimulated a large region of bone growth, while bone trauma resulted in a localized bone response as indicated by jagged bone at the linea aspera. However, it was not until the combinatory factors were included that an HO structure similar to that of humans formed more readily in 60% of the sheep. In conclusion, data suggested that traumatic HO growth can develop following various traumatic factors, but a combination of known instigators yields higher frequency size and consistency of ectopic bone.

## 1. Introduction

Heterotopic ossification (HO) is an abnormal bone growth that occurs outside the skeleton within the adjacent soft tissues [1,2]. The direct cause of HO is debatable; however, it has been commonly categorized in three distinct forms: (1) traumatic HO, which can be induced by bone/soft tissue trauma and/or burns, (2) neurogenic HO, which appears following spinal and/or head injuries, and (3) genetic/hereditary HO, due to rare disorders [3,4,5]. Traumatic HO is frequently observed in Service Members following combat-related trauma. Estimates suggest that ~65% of Service Members who suffer limb loss or major extremity trauma experience some type of HO formation [6,7,8,9]. Radiographic and histological evaluation of HO show that its mature state includes cancellous bone encased by a cortical shell [10]. More specifically, traumatic HO is similar to cancellous bone with slight, but important, differences where the bone exhibits a complex hybrid trabecular-like bone structure with osteon remodeling, as well as hyper-mineralized regions of woven bone [11,12,13,14]. Traumatic HO can be both extra-skeletal or stem from the periosteum of an amputee’s resected limb [15,16]. HO development delays rehabilitation, particularly due to the pain associated with exercise or prosthetic socket use. Ectopic bone that develops in soft tissues can cause tenderness and even debilitate movement, thus requiring modifications to prosthetic limb componentry and socket size, or prevents their use altogether [17,18]. A main concern/limitation regarding how to treat and prevent HO is a lack of accurate translational animal models that identify the direct cause of HO formation.

Recent studies by our team demonstrated that traumatic HO growth, similar to that observed in humans with limb loss, is conceivable using a large animal model following a combination of combat-relevant trauma [19]. Within that study, we demonstrated that 65% of sheep (5 out of 8) developed the aforementioned hybrid traumatic HO bone structure, similar to what is observed clinically. Although our study exhibited HO formation, we did not elucidate which trauma factor contributed most to the observed pathology. Therefore, in this study, we performed large animal work wherein various combinatorial trauma factors were used to more clearly delineate the contributing factors that trigger HO, with a hypothesis that medullary canal/bone marrow leakage/exposure from bone trauma would be the greatest contributor [8,12,20,21,22].

## 2. Materials and Methods

### 2.1. Supplies, Instruments, and Reagents

Surgical supplies, Petri dishes, agar, and brain heart infusion (BHI) broth were purchased from Fisher Scientific (Waltham, MA, USA). The University of Utah Comparative Medicine Center provided any fluids, analgesics, and anesthetics required. The Bone and Biofilm Research Lab. (Salt Lake City, UT, USA) provided surgical tools and histological processing equipment/materials. Martin Engineering donated the Martin^®^ Tornado Air Cannon (Model BB4-12-28, Martin Engineering, Neponset, IL, USA). The V.A.C. Freedom^®^ Therapy Units were purchased from KCI (San Antonio, TX, USA). Calcein green was purchased from Sigma Aldrich (St. Louis, MO, USA). Silica (Si) beads were purchased from Fisher Scientific (Waltham, MA, USA). Micro-CT scans were captured using a Quantum GX micro-CT (PerkinElmer; Waltham, MA, USA). Scanning electron microscopy was performed on a JEOL JSM-6610 (JEOL, Peabody, MA, USA). Sanderson’s Rapid Bone Stain (SRBS) was purchased from Dorn & Hart Microedge, Inc. (Loxley, AL, USA).

### 2.2. Air Impact Device

To replicate the effects of an improvised explosive device (IED) and/or rocket propelled grenade (RPG) blast, which has been linked to the formation of HO in wounded warriors, we utilized a Martin^®^ Tornado Air Cannon with a four-inch valve. The parameters of the air blast system were previously optimized and defined for this animal model [19,23]. In brief, the air impact device (AID) delivers a rapid depressurization of air within 0.1 s. The estimated incident pressure reaches approximately 588 kPa for a 100 PSI blast, consistent with conditions that may be encountered in a battlefield blast scenario, as determined by parameters from the Kingery–Bulmash blast parameter calculator.

### 2.3. Biofilm Growth

**Figure 1:**
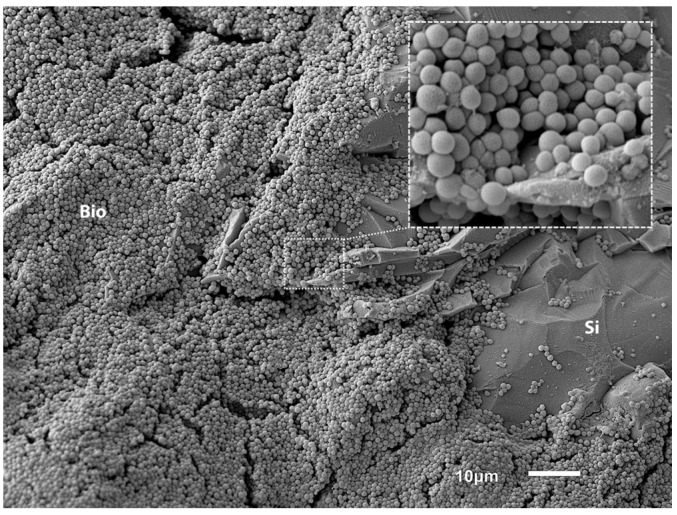
Representative SEM image showing *S. aureus* ATCC 6538 biofilm formation on a roughened Si bead following ~72 h of growth. Note the multi-layer biofilm (bio) across the roughened Si surface. Higher magnification (box) demonstrates the biofilm to be a multicell layer structure.

In a similar manner as in our previously published model, mature biofilms of *Staphylococcus aureus* ATCC 6538 were grown on Si beads (~3 mm in diameter) to be used as initial inocula for groups 2, 3, and 7 (groups defined below) [19]. In short, 60-grit sandpaper was used to roughen the Si beads, thus increasing the surface area and improving bacterial adherence/biofilm formation. Biofilms matured under shaking on an orbital shaker (40 rpm). Fresh BHI was reloaded every 24 h until a total of ~72 h growth was achieved. Five Si beads were selected at random from each batch to inoculate sheep in groups 2, 3, and 7. The remaining beads were analyzed for either the colony forming units (CFU)/bead or to perform secondary electron imaging (SEI) to evaluate the biofilm morphology on the Si bead surface (Figure 1).

### 2.4. Animal Model and Trauma Factors

Animal work was performed with approval and oversight of the University of Utah’s Institutional Animal Care and Use Committee (IACUC) and the United States Army Medical Research and Development Command Office of Research Protections (USAMRDCORP) Animal Care and Use Review Office (ACURO). The surgical approach was previously described [19]. In brief, skeletally mature, female Rambouillet sheep (age 1–3 years) were purchased from K Bar Livestock (San Antonio, TX, USA) and randomly assigned to a group until all groups contained an equal number of sheep. The evening prior to the AID blast and surgical procedure, a fentanyl patch was applied. Sheep were sedated with an intravenous (IV) injection of Propofol (3–7 mg/kg) and intubated. The limb was prepared using standard surgical prep techniques, an incision was made in the midshaft region of the right femur through the deep tissue to expose the bone. Depending on the experimental group (see Table 1), surgeons applied various of the combinatorial factors to simulate trauma that would be relevant to an injured Service Member. These individual, simulated factors consisted of the following:

1. AID: The AID was positioned above the incision site, pressurized to 100 PSI, and discharged. This was repeated five times. Fluoroscopic images were captured to confirm the AID blast did not cause bone fracture, as required by the IACUC, to ensure weightbearing.
2. Bone Trauma (BT)
  - Periosteal Disruption (PD): The outer cortical bone surface was roughened/feathered using a periosteal elevator and osteotome, simulating bone trauma that may accompany a blast-related injury.
  - Transcortical Defect—Drill Holes (DHs): In addition to bone roughening, three 5 mm defects (drill holes) were equidistantly created along the axis of the femur midshaft, allowing for bone marrow/growth factors to be released into the adjacent muscle.
  - Bone Fragments (BFs): A bone core was collected from the distal femoral condyle (through the same incision in the midshaft of the femur), then fragmented using a rongeur. The bone fragments/sample were mixed with saline to create a “bone slurry” that was applied to the disrupted area of the femur, simulating bone fragments and host medullary canal components that would be present in a blast-related injury.
3. Biofilm (Bio): Sheep were inoculated with *S. aureus* ATCC 6538 biofilms. To inoculate, the incision line was reopened following the AID blasts and five beads that contained mature biofilms were placed adjacent to the midshaft of the femur within the muscle.
4. Tourniquet (T): A tourniquet was placed for 45 min to simulate battlefield injury protocol.
5. Negative Pressure Wound Therapy (NPWT): NPWT was applied subdermally at a consistent negative setting of 175 mmHg for a duration ranging from 3 to 7 days. The foam used in the therapy was removed within ~24 h of fluid cessation in the external canister. Sheep in Groups 3 and 4 required a 2nd surgery to remove the NPWT foam.

### 2.5. Post-Surgical Monitoring and Calcein Green Labeling

Following surgery, sheep were recovered and resumed weightbearing activity. Sheep were closely monitored twice daily for any signs of pain or distress. Sheep displaying such signs were promptly treated with Buprenex (0.005–0.01 mg/kg) and/or additional fentanyl patches. Each sheep received injections of calcein green (10 mg/kg) on two separate occasions. Solution preparation was described previously [19]. The first injection was administered intravenously 16 days before euthanasia, while the second occurred 5 days prior to euthanasia. These two injections of calcein green allowed for the assessment of bone viability and determined the rate at which bone re/modeled. HO has been shown to predominantly occur within 3 to 12 weeks after injury; however, it can take up to 6 months to manifest [24]. Sheep were humanely euthanized 24 weeks post-surgery with an injection of Beuthanasia D (1 mL/4.5 kg). A total of *n* = 35 of the sheep from the 7 study groups reached their pre-determined endpoint, while *n* = 1 was euthanized early due to a broken leg that was unrelated to the surgery (Table 1).

### 2.6. Microbiology, Radiography, and Micro-CT

Following euthanasia, sheep that were inoculated with biofilm (Groups 2, 3, and 7) were prepped to aseptically obtain subdermal samples and determine if bacteria were still present following the 24-week timepoint. Tissue and bone core samples were collected using a biopsy needle (Jamshidi™) in the approximate region where biofilms (on Si beads) were inoculated. As CFUs were not the primary outcome measure to determine HO formation, samples were collected adjacent to the inoculation site to ensure that potential HO was not disrupted. Tissue/bone samples were weighed, added to 1 mL of PBS, ground in a tissue grinder, vortexed/sonicated, and plated using a 10-fold dilution series to quantify CFU/g tissue.

Following tissue collection, the full hind limbs were harvested, assigned a blinded laboratory accession number, and radiographed. The tibia and patella were disarticulated, and the superficial tissue was removed from the femur, allowing for the whole femur to fit within the micro-CT chamber. Micro-CT scans were captured with a tube voltage of 90 kVp, tube current of 180 μA, and a field-of-view (FOV) of 75 mm, resulting in slices with a thickness of 148 µm. The scans were processed using 3D Slicer to determine the presence of ectopic bone [25]. Additionally, the micro-CT scans were imported into Seg3Dto determine the volume of bone outside the cortical boundary of the periosteum.

### 2.7. Histological Processing

Additional soft tissue was dissected around the femur, allowing for 3 cm sections to be captured midshaft of where the surgical trauma occurred to ensure proper specimen fixation in 10% neutral buffered formalin. The specimens were dehydrated in ethanol, infiltrated, and embedded in poly-methyl-methacrylate (PMMA) using our standard techniques [19,26,27]. Following polymerization, the samples were sliced transversely using a water-cooled saw equipped with a diamond-coated blade, resulting in sections ~2–3 mm thick. These sections were then ground and polished to an optical finish using a variable-speed grinding wheel. A conductive layer of carbon was applied to the polished surface of the specimens for ~30 s prior to imaging with a SEM equipped with a backscatter electron (BSE) detector and accompanying JEOL software v1.0 [28]. Following imaging, the digital images were processed using Microsoft Research Image Composite Editor (MRICE; v2.0.3.0) to mosaic/stitch BSE images together, creating an overhead view for analysis.

### 2.8. Mineral Apposition Rate and Light Microscopy

Following SEM imaging, the specimens were further processed by removing the thin layer of carbon, and then adhered to plastic slides using an EXAKT 402 Precision Adhesive Press (EXAKT Technologies, Oklahoma City, OK). Slides were then ground to a final thickness of ~75 µm and viewed under a mercury lamp microscope to visualize the fluorochrome double-labeled bone. The average thickness of the newly mineralized bone was calculated and expressed in µm/day [12,29]. Mineral apposition rate (MAR) was calculated using the following equation:

MAR [μm/day] = [∑x €(π/4)]/nt(1)

where ∑x = sum of measurements between double labels, € = micrometer calibration factor (microns), π/4 = obliquity correction factor, n = number of measurements, and t = time (days).

Light microscopy was conducted following MAR analysis by staining each section with SRBS [30]. The stained sections were visually examined for evidence of newly-formed osteoid and the presence of osteoblasts.

### 2.9. Statistical Analysis

Outcome measures of the MAR analysis were compared statistically using independent *t*-tests with an alpha level of 0.05.

## 3. Results

### 3.1. Biofilm Growth, NPWT, and Animal Monitoring

As previously published, the use of roughened Si beads resulted in mature biofilm formation [19]. Confirmation through SEI analysis revealed uniform coverage across the surface of the Si beads (Figure 1). Microbiological quantification indicated that, on average, each Si bead contained ~7 × 10^6^ CFU, resulting in a total of ~3.5 × 10^7^ CFU for five Si beads, which constituted the inoculation amount for each animal. The “wrapped” NPWT foam proved to be unproblematic and allowed for the continuous subdermal application of negative pressure.

Regardless of the group, the sheep tolerated the AID blast and surgical trauma with minimal signs of distress. Fevers were never present and the sheep did not require antibiotics. The majority of sheep were lame and limped for approximately 1 week post-procedure. Limping was more prevalent in groups that utilized NPWT. Sheep with biofilm only and/or AID blast only showed minimal reaction to the surgical trauma. One contingency sheep was needed throughout the course of the study as it suffered a bone fracture unrelated to the surgery.

### 3.2. Microbiology, Radiography, and Micro-CT

**Figure 2:**
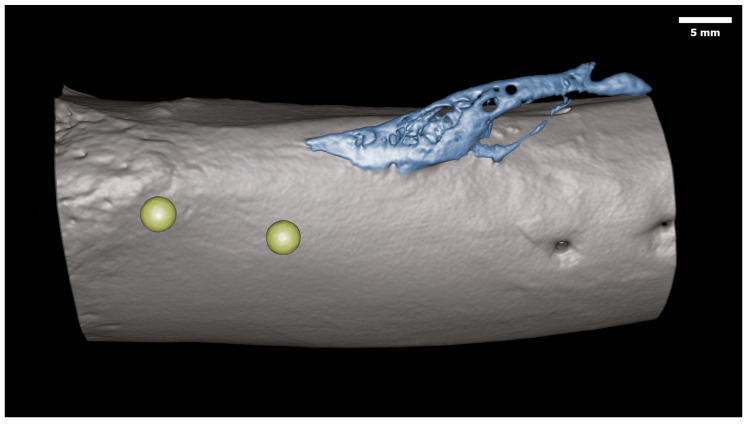
Representative three-dimensional reconstructed micro-CT image demonstrating volume analysis on a scan from Group 7. Note the ectopic bone (blue) stemming from the posterior side of the femur. Si glass beads (yellow) used to grow biofilms were observed in the adjacent muscle.

Microbiological data showed little to no bacterial growth in the muscle and/or bone regions that were sampled for Groups 2, 3, and 7, with no obvious signs of infection. Radiography was deemed insufficient to accurately determine the structure of the ectopic bone due to the projection effect. Micro-CT scans provided a detailed overview of the bone surface, which revealed ectopic bone stemming from the posterior side in select groups. More specifically, the micro-CT analysis demonstrated that the combination of traumatic factors in Group 7 (all trauma and surgical-related factors excluding NPWT, which was previously published by our team) [19], demonstrated the most consistent bone response (5 out of 5) by 24 weeks. A reactive bone response was also observed in the biofilm and bone trauma groups, Group 2 (2 out of 5), Group 3 (4 out of 5), Group 5 (5 out of 5), and Group 6 (5 out of 5) sheep. No obvious signs of ectopic bone were detected in Groups 1 or 4 by way of micro-CT (Table 2). On the sheep where a bone response did occur, a similar volume of bone was present (~6 mm^3^) outside the cortical boundary of the periosteum on the shaft of the femur, regardless of the group. This similarity was due to the inability of the micro-CT scans to distinguish the difference between a periosteal reaction, biofilm response, and traumatic HO, which resulted in measuring artifacts. No growth/bone response was observed on the contra-lateral limb, suggesting the trauma and AID blast were localized only to the surgically operated limb. Due to the limited resolution of the micro-CT scans, only areas with a significant bone response could be observed (Figure 2). More definitive outcomes were provided by BSE imaging.

**Figure 3:**
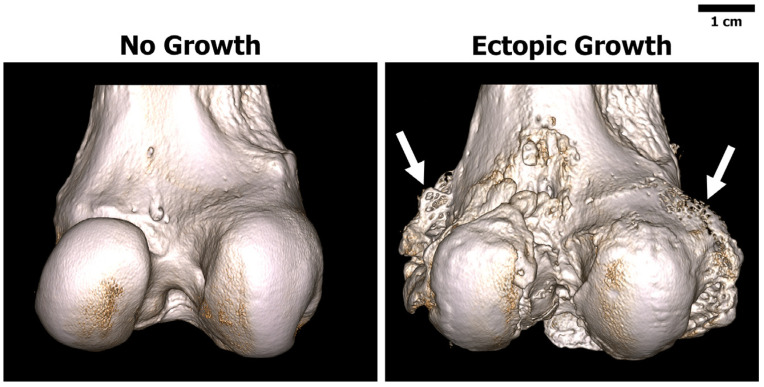
Representative three-dimensional reconstructed micro-CT scans of the distal femoral condyles. (**Left**) Sheep from Group 5 showing no signs of bone growth. (**Right**) Sheep from Group 7 showing ectopic bone growth (arrows) stemming from the medial and lateral sides. Note this response was only observed in Group 7 (2 out 5 Sheep).

Group 7 demonstrated reactive bone growth on 40% of the distal condyles (2 out of 5) stemming from the medial and lateral sides (Figure 3). This growth appeared to be unrelated to the bone cores that were collected to produce the bone chip slurry. This observed distal growth was not observed in the other six groups.

### 3.3. Scanning Electron Microscope

**Figure 4:**
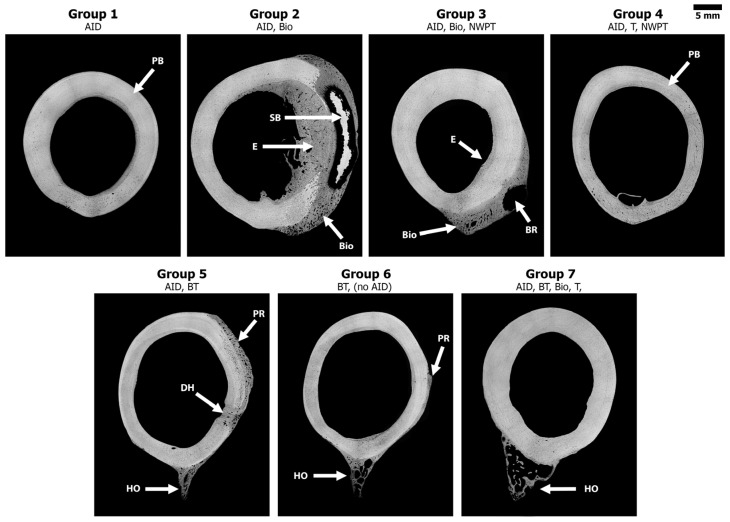
Representative SEM BSE images (stitched using MRICE to create an overhead cross-sectional view) demonstrating the various bone responses observed in each group. Grey = bone and black = soft tissue/pore space. PB = Plexiform Bone, E = Endosteal Thickening, SB = Sequestrum Bone, Bio = Biofilm-Induced Bone, PR = Periosteal Reaction, DH = Drill Hole, HO = Ectopic Bone. Note the biofilm groups (2 and 3) showed a large volume of new bone, while the bone trauma groups (5 and 6) demonstrated the jagged bone response. Group 7 reveled a mature HO structure similar to what is observed in human HO. Groups 1 and 4 remained relatively unaltered.

The superior resolution of the BSE images enabled more accurate analysis of the varied bone response. Select sheep from Groups 2, 3, 5, 6, and 7 demonstrated a significant bone response. However, the level of bone response and complexity of the structure varied from sheep to sheep within each group (Figure 4). Overall, the anterior and medial regions revealed that the bone was still in its native plexiform structure for all groups, which suggested that the various trauma factors were localized to the specific region. Minimal to no bone response was observed in Groups 1 and 4, the groups which contained no biofilm or bone trauma. This suggested that the AID and NPWT alone did not result in a significant bone response, only a soft tissue response.

When the Si biofilm beads were in close approximation to the bone in Groups 2 and 3, the analysis revealed an aggressive bone reaction that did not follow a classic infection response. The analysis of those groups also demonstrated regions of sequestrum bone, endosteal thickening, and extensive new bone growth extending from the periosteum into the surrounding tissue that did not resemble the traumatic HO bone structure. The combination of biofilm and trauma appeared to cause multiple complex bone responses.

**Figure 5:**
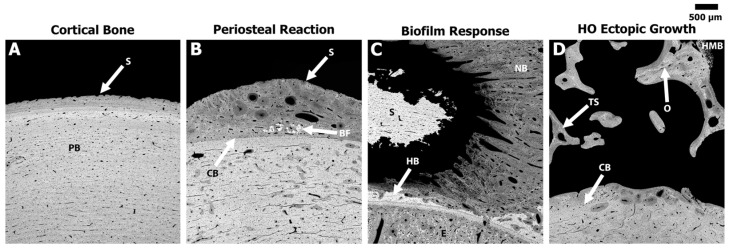
Representative high magnification BSE images of the different bone responses observed due to individual/combination of the various surgical/trauma factors. Grey = bone and black = soft tissue/pore space. (**A**) Native sheep cortical bone from the anterior regions that remained in the plexiform (PB) state presenting no significant response associated with the AID blast. Note the smooth (S) surface of the circumferential lamellae. (**B**) Periosteal reaction (darker grey) extending from the cortical boundary (CB) on the lateral side of the femur where bone feathering/roughening occurred. Note the smooth/dense (S) nature of the periosteal reaction in this region as well as the incorporated bone fragments (BF). (**C**) Bone response due to the inoculated biofilm showing an extensive amount of new bone (NB) growth as well as endosteal (E) thickening adjacent to the sequestrum (S) bone. Note the host bone (HB) is almost completely resorbed/remodeled adjacent to the Si beads. (**D**) HO ectopic bone growth showing a complex hybrid bone structure that resembles traumatic HO observed in humans. Note the ectopic bone demonstrated a trabecular-like structure (TS) with osteon (O) remodeling, and areas of hyper-mineralized woven bone (HMB) extending from the surface.

The lateral region in Groups 5 and 6 where the bone was roughened/feathered, demonstrated osteonal remodeling mid-cortex along with a smooth periosteal response extending ~1–2 mm from the outer boundary of the cortical bone. The transcortical 5 mm defects (drill holes) demonstrated an osseous union that bridged the 5 mm defect with minimal external growth within the canal or outside the cortical boundary (Figure 4). Lastly, the high-resolution BSE analysis demonstrated that 3 out of 5 sheep from Group 7 (all trauma factors, excluding NPWT) exhibited ectopic bone that resembled human traumatic HO. This HO ectopic bone growth appeared to be a complex hybrid trabecular-like structure with osteon remodeling as well as hypermineralized regions. This observed bone growth differed from the smooth periosteal and/or biofilm responses that were predominate in the other groups (Figure 5). One sheep each from Groups 3, 5, and 6 also demonstrated this HO response, but it was minimal compared to the HO response in Group 7 (Table 2).

### 3.4. MAR and Light Microscopy Analysis

Fluorochrome label analysis demonstrated minimal remodeling in Groups 1 and 4, confirming that AID and/or NPWT alone did not trigger a significant bone response. In contrast, Groups 2 and 3 demonstrated that when the Si biofilm beads were in close approximation to the bone, significant areas of bone loss/resorption occurred. Within these resorbed regions, extensive amounts of new bone were observed forming a sheet-like layering structure of fluorochrome labels, indicating a biofilm-infected bone response as described previously [19].

**Figure 6:**
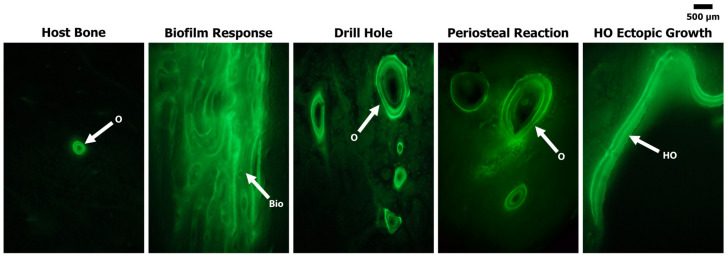
Representative images showing the different bone remodeling structures by way of fluorochrome double labels (green). Note the host bone, drill hole and periosteal reaction labels were circular osteonal double labels (O), while the biofilm response (Bio) demonstrated aggressive re/modeling near the previously resorbed host bone. The HO ectopic bone modeling (HO) was similar to trabecular remodeling, with linear double labels predominantly at the bone seams.

**Figure 7:**
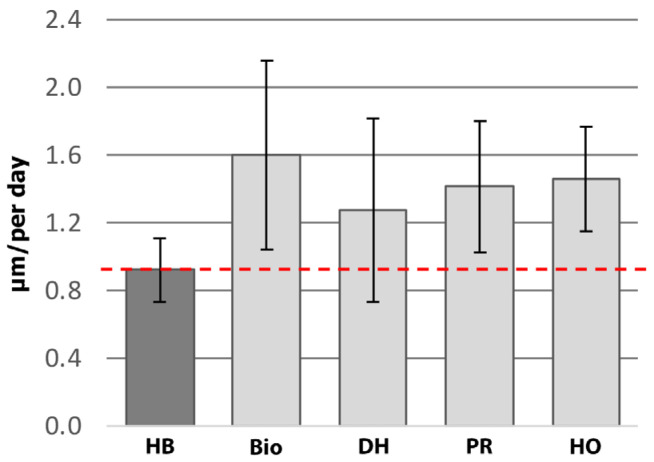
Double-label analysis showing an increased MAR in all bone responses compared to the host bone (red dotted line). HB = Host Bone, Bio = Biofilm-Induced Bone Response, DH = Drill Hole, PR = Periosteal Reaction, HO = Heterotopic Ossification.

Groups 5, 6, and 7 revealed standard osteon remodeling in the unaltered mid-cortex region of the cortical bone, within the periosteal response, in the osseous unions of the 5 mm defects and at the linea aspera interface. In select regions where ectopic bone that resembled human HO occurred, the fluorochrome labeling demonstrated distinct double labels similar to those of trabecular modeling, which was in stark contrast to the standard circular osteonal remodeling observed across all other regions of new bone growth, with the exception of the biofilm infection response (Figure 6). The fluorochrome analysis also revealed an increase in MAR in all the various types of bone responses: biofilm (1.6 ± 0.6 μm/day), periosteal reaction (1.4 ± 0.4 μm/day), drill holes (1.3 ± 0.6 μm/day), and HO ectopic bone (1.5 ± 0.3 μm/day), compared to the unaltered host cortical bone (0.9 ± 0.2 μm/day) (Figure 7). However, no statistical significance was observed when comparing the MAR of the biofilm reaction (*p* = 0.5923), periosteal response (*p* = 0.7868), and drill holes (*p* = 0.4562) to the ectopic bone that resembled human HO. Light microscopy analysis confirmed the presence of osteoblasts and osteoid at the fluorochrome-labeled regions, supporting the previous SEM and MAR analyses showing that the bone was still actively modeling/healing from the multiple traumatic factors 24 weeks post-op.

### 3.5. Main Findings

Overall, the histological analyses demonstrated that the presence of mature biofilm stimulated a large region of bone growth when placed in close proximity to the bone, while the bone trauma resulted in a localized bone response that had the ability to induce jagged bone at the linea aspera. When combining the trauma factors (minus NPWT, Group 7) a structure similar to human HO was observed in 60% of the sheep (Table 2).

## 4. Discussion

HO continues to remain a major concern with traumatic injuries, in particular blast injuries to Service Members that result in limb loss. The lack of a clinically relevant animal model compounds this problem, as research cannot be advanced to understand new preventive measures. Our team recently replicated what is observed clinically, with 5 out of 8 (63%) sheep exhibiting a hybrid HO bone structure [19]. While promising, that study contained a combination of five different traumatic factors, but did not elucidate which traumatic factor was the most prevalent in triggering HO growth. Nevertheless, the study did confirm that traumatic HO bone may be accurately identified by way of BSE analysis, revealing the hybrid bone morphology with a trabecular-like bone structure, the presence of osteon remodeling, and varying degrees of mineralization.

In this ovine model, we sought to observe effects of individual and layered/combinatorial traumatic factors and their influence on HO growth. The AID, tourniquet, and NPWT revealed no obvious signs of a bone response when tested unaccompanied by other traumatic factors. This suggested that soft tissue damage alone does not trigger HO growth in a large animal model of blast-related trauma. The biofilm groups, absent of any bone trauma, demonstrated that if/when the inoculated biofilm was in close approximation to the bone, an extensive response was observed. Rather than simply resulting in necrotic, infected bone, the biofilm triggered an extensive localized response with a large amount of new bone growth, extending past the cortical boundary. Although infection was clearly present, the biofilm had minimal effect on animal well-being, indicating that biofilms as initial inocula produced a low-lying, quiescent infection that differs from a raging bone infection. When the biofilm was paired with NPWT, the chances of observing a bone response doubled; however, this observation was more likely due to the proximity of the Si biofilm beads.

The periosteal disruption and transcortical defect from the BT groups (5, 6, and 7) revealed two separate bone responses. First, the disrupted site always triggered a bone response. The response was predominantly a standard smooth periosteal reaction and/or an osseous union of the drill holes. However, select sheep demonstrated jagged bone growth stemming from the posterior side near the linea aspera, although this response was not consistent between sheep. The addition of the AID to the bone trauma did not appear to drastically change the bone response. Lastly, when combining the periosteal disruption and AID with biofilm and tourniquet placement, new bone growth that resembled traumatic HO was observed 60% of the time. This suggested that increased trauma with multiple factors may be required to consistently stimulate HO growth.

Fluorochrome data indicated an increased MAR in all the various bone response types compared to the unaltered host bone. This increase in the remodeling rate suggested that the trauma-induced bone response was still in the active healing/modeling phase 24 weeks post-op. It is unclear whether HO would continue to grow and/or develop following the 24-week timepoint. Fluorochrome labels further validated the unique bone responses observed by way of BSE imaging. The biofilm response and the ectopic HO growth both demonstrated unique modeling patterns, with sheet-like layering and linear double labels at the bone seams, respectively. This was in stark contrast to all other regions, which contained the standard circular osteonal remodeling.

The limitations of this study include the lack of bone fracture and amputation (which has demonstrated increased HO formation in rodents) [31] and thermal injures (i.e., burns) [32], and the fact that the localized AID blast does not influence brain injury. These limitations might help explain the low volume of HO exhibited compared to what would be excised from injured Service Members. Additionally, just seven groups were tested, rather than all individual/combinatorial factors. Multiple timepoints may also have been beneficial to observe the early bone response as well as to determine if HO continues to form past the 24-week timepoint. Additional studies are currently ongoing (which include an amputation model with an open medullary canal) to address some of these limitations, to continue advancing the model and determining if the HO bone volume increases.

## 5. Conclusions

In summary, the analyses from this study demonstrated that the presence of mature biofilm stimulated a large region of bone growth, while bone trauma resulted in a localized bone response that had the ability to induce jagged bone at the linea aspera. When combining the trauma factors, a structure similar to human HO was observed to a much greater degree (in 60% of the sheep; Group 7). The outcomes suggested that traumatic HO growth is extremely complicated and has the ability to develop with an individual traumatic factor but, most likely, a combination of traumatic factors leads to more consistent and severe HO formation.

## Figures and Tables

**Table 1:** Number of sheep and surgical induced trauma/procedure(s) performed for each group.

Groups	# Animals per Group	Timepoint	Surgical Trauma
1	*n* = 5	24 Weeks	AID
2	*n* = 5	24 Weeks	AID and Bio
3	*n* = 5	24 Weeks	AID, Bio, and NPWT
4	*n* = 5	24 Weeks	AID, T, and NPWT
5	*n* = 6 ^1^	24 Weeks	AID, and BT
6	*n* = 5	24 Weeks	BT (no AID)
7	*n* = 5	24 Weeks	AID, BT, Bio and T

NOTE: *n* = 1 sheep from Group 5 was euthanized early. ^1^ AID = Air Impact Device, BT = Bone Trauma, Bio = Biofilm, T = Tourniquet, and NPWT = Negative Pressure Wound Therapy.

**Table 2:** Summary of all analyses demonstrating that the combination of multiple trauma/surgical factors resulted in a higher chance of observing ectopic bone similar to traumatic HO.

Groups	Surgical Trauma	Demonstrated a Bone Response	Ectopic Bone Distal Condyle	Confirmed HO Growth
1	AID	-	-	-
2	AID and Bio	40%	-	-
3	AID, Bio, and NPWT	80%	-	20%
4	AID, T, and NPWT	-	-	-
5	AID, and BT	100%	-	20%
6	BT (no AID)	100%	-	20%
7	AID, BT, Bio and T	100%	40%	60%

AID = Air Impact Device, BT = Bone Trauma, Bio = Biofilm, T = Tourniquet, and NPWT = Negative Pressure Wound Therapy.

## Data Availability

The original contributions presented in the study are included in the article, further inquiries can be directed to the corresponding author/s.
